# WHO Non-Lab-Based CVD Risk Assessment: A Reliable Measure in a North Indian Population

**DOI:** 10.5334/gh.1148

**Published:** 2022-09-01

**Authors:** P. Ananda Selva Das, Mahasweta Dubey, Ravneet Kaur, Harshal Ramesh Salve, Cherian Varghese, Baridalyne Nongkynrih

**Affiliations:** 1 Centre for Community Medicine, All India Institute of Medical Sciences, New Delhi, India; 2 Room. 14, Old OT block, Centre for Community Medicine, All India Institute of Medical Sciences, New Delhi, India; 3 Room. 18, Old OT block, Centre for Community Medicine, All India Institute of Medical Sciences, New Delhi, India; 4 Room. 12, Old OT block, Centre for Community Medicine, All India Institute of Medical Sciences, New Delhi, India; 5 Department of Noncommunicable Diseases, World Health Organization, Geneva, Switzerland

**Keywords:** Non-communicable diseases, cardiovascular disease risk, WHO CVD risk charts, risk prediction, agreement, risk factors

## Abstract

**Introduction:**

Timely, affordable, and sustained interventions reduce the risk of heart attack or Stroke in people with a high total risk of cardiovascular diseases (CVD). Risk prediction tools are available to estimate the cardiovascular risk using information on multiple variables. CVD risk charts prepared by the World Health Organization (WHO) has laboratory-based and non-laboratory-based charts with the latter meant for use in resource limited settings. We conducted a study to determine concordance between the laboratory- and non-laboratory risk charts and to estimate the prevalence of selected CVD risk factors in a rural Indian population.

**Methods:**

A community-based cross-sectional study was conducted in rural area of Ballabgarh in district Faridabad, Haryana. Sample of 1,018 participants aged 30–69 years was selected randomly from study area. Information on CVDs risk factors was obtained using WHO STEPS questionnaire, anthropometry and laboratory investigation. Risk distribution among the study participants was observed. Concordance between laboratory- and non-laboratory-based WHO CVD risk charts was determined using agreement analysis.

**Results:**

The mean age of the study participants was 43.9 (8.9) years and 55.6% participants were women. Among various CVD risk factors, hypertension (39.4%) was the major factor followed by overweight (34.1%) was found to be major factor, followed by current smoking (23.6%) and hypercholesterolemia (18.7%). The concordance between the two charts was 83.3% with kappa value of 0.64. Considering laboratory-based charts as the gold standard, the sensitivity and specificity of non-laboratory-based risk charts at 5% risk as cut-off was 86.5% and 90.3% respectively.

**Conclusion:**

The study shows a good agreement between the laboratory-based and non-laboratory-based risk charts. Thus non-laboratory-based risk charts are suitable for risk estimation of CVDs for use in resource limited settings like India.

## 1 Introduction

Cardiovascular diseases (CVDs) are leading cause of mortality and morbidity globally. Ischemic heart disease and stroke are responsible for the majority of deaths due to CVDs [1,2]. CVDs are caused by an interaction of multiple risk factors which include tobacco use, harmful alcohol use, physical inactivity, unhealthy diet, high blood pressure, high blood glucose, high body mass index (BMI) and high blood cholesterol. Individual level attributes such as age, sex, ethnicity/race, and family history of cardiovascular disease add to overall risk [2]. Identification and addressing risk factor at right time among individual with high risk would be helpful in the prevention of CVDs [3]. Risk prediction charts help to quantify CVD risk by considering multiple risk factors and categorize individuals into high and low risk. Timely and affordable interventions will reduce the risk of severe outcomes such as heart attack, and hence will reduce premature mortality and disability. These CVD risk charts are also good communication tools for monitoring behavior change at individual level [3,4].

Framingham Risk Score (FRS) was the first CVD risk prediction charts which was developed based on the Framingham Heart Study. Later, multiple risk prediction charts were developed and are in use (examples include SCORE, PROCAM, QRISK, Reynold Risk Score etc) [5,6,7,8]. But most of these charts are based on the studies in high-income countries and may not be useful to resource limited setting [9]. The World Health Organization (WHO) in association with International Society of Hypertension (ISH) developed a risk prediction chart in 2007 which is applicable to 14 WHO epidemiological regions. WHO updated the chart with two versions viz, laboratory-based and non-laboratory-based in year 2019 with wider usability. The laboratory-based WHO CVD risk charts require information on age, sex, smoking status, systolic blood pressure, diabetes, and total cholesterol values to estimate the CVD risk in an individual. Risk stratification is done as follows: <5% (green), 5% to <10% (yellow), 10% to <20% (orange), 20% to <30% (red), and ≥30% (dark red). This risk stratification is aligned with the WHO recommendations for management of CVD risk. However, in resource constraint settings where either availability or accessibility of laboratory investigations is limited these charts are of no use. In such situation, non-laboratory-based risk charts using information such age, sex, smoking status, systolic blood pressure and BMI, is an effective tool for CVD risk assessment. Non-laboratory-based CVD risk charts are also useful for field level workers for screening and referral of individuals at risk of CVD in the community [5,10]. Risk stratification is similar to lab-based charts, however its validity for use in resource limited setting like India is not yet studied. Hence, we aimed to estimate the concordance between laboratory based and non-laboratory based WHO risk charts.

## 2 Methods

This was a community based cross-sectional study conducted from March 2020 to January 2021. The study was carried out in rural villages under Comprehensive Rural Health Services Project (CRHSP), Ballabgarh in Faridabad district of Haryana in North India. The study area is a health and demographic surveillance site and all demographic and health information are stored in a computerized Health Management Information System (HMIS) [11]. Two villages were selected for the study from the area. The sample size was calculated based on the prevalence of diabetes (9.3%) reported in National Noncommunicable Diseases Monitoring Survey 2017–2018 (NNMS 2017), with 2% absolute precision and 10% non-response, where the minimum sample size required was 1000 [12]. Individuals in the age group of 30–69 years in the study villages were enlisted from the HMIS and a simple random sampling was done to select the participants. Participants with history of event of stroke or myocardial infarction were excluded.

A semi-structured questionnaire, adapted from the WHO STEPS questionnaire, was used to collect information about CVD risk factors. Anthropometric measurements (height and weight), assessment of blood pressure, random blood glucose and lipid profile (total cholesterol, triglycerides, high density lipoprotein and low-density lipoprotein) were carried out using standard procedures and equipment.

The updated WHO CVD risk charts for South Asia, both laboratory-based and non-laboratory-based charts were used to estimate the CVD risk among the participants. The risk charts give CVD risk in percentages and has been further categorized into 5 groups –<5%, 5–<10%, 10–<20%, 20–<30% and ≥30%.

All current smokers and those who quit smoking less than one year before the assessment were considered smokers; *Hypercholesterolemia* was defined as total cholesterol value of ≥ 200 mg/dL; *Body Mass Index (BMI)* was calculated by the ratio of weight in kilograms divided by square of height in meters. Subjects were classified as normal if the BMI was 18.5–24.99 kg/m^2^, overweight if 25–29.99 kg/m^2^ and obese if BMI ≥ 30 kg/m^2^; *High blood pressure* was defined as systolic blood pressure ≥ 140 mm Hg and/or diastolic blood pressure ≥ 90 mm Hg; *Hypertension* was defined as systolic blood pressure ≥ 140 mm Hg and/or diastolic blood pressure ≥ 90 mm Hg or a history of taking anti-hypertensive medications or supported by documentation; Raised blood glucose was defined as random blood glucose ≥ 200 mg/dL; *Diabetes mellitus* was defined as random blood glucose ≥ 200 mg/dL or a history of taking anti-diabetic medications or supported by documentation.

Statistical analysis: The data was collected digitally using Google forms. The collected data was extracted to Microsoft Excel and was analyzed using STATA version14. Categorical variables were expressed in percentages. Statistical significance between various categorical variables and gender was assessed using Chi-square test and Fischer’s exact test, wherever applicable. Concordance and non-concordance between the laboratory-based and non-laboratory-based charts was calculated assuming the lab-based charts as reference. If the CVD risk calculated by non-lab-based risk charts was higher than the risk calculated by lab-based risk charts, it was described as overestimate and if lower, underestimate. Agreement between the two charts was determined by Cohen’s kappa statistic. Sensitivity and specificity of non-laboratory-based risk charts were calculated with laboratory-based risk charts considered as gold standard (reference).

Ethical approval was obtained from the Institutional Ethics Committee, All India Institute of Medical Sciences, New Delhi. (Ref. No.: IECPG-292/22.07.2020, RT-10/26.08.2020) Informed written consent was taken from all the participants.

## 3 Results

A total of 1,018 participants were enrolled in the study. The Mean (SD) age of the study participants was 43.9 (8.9) years. Majority (N, 70.3%) of the study participants were in the age group of 30–49 years while only 2.9% in 60–69 years. Female participants (N, 55.6%) were slightly more than the male participants (44.4%) (Table 1).

**Table 1:** Distribution of participants by age & sex and prevalence of CVD risk factors by age groups (N = 1018).


AGE GROUP	30–39 n (%)	40–49 n (%)	50–59 n (%)	60–69 n (%)	TOTAL n (%)

**Gender**					

**Male**	142 (31.4)	168 (37.2)	129 (28.5)	13 (2.9)	452 (44.4)

**Female**	218 (38.5)	188 (33.2)	143 (25.3)	17 (3.0)	566 (55.6)

**Total**	360 (35.4)	356 (35)	272 (26.7)	30 (3.0)	1018 (100.0)

**CVD RISK FACTORS**	**% (95% CI)**				

**Current smoking**	10.6 (7.8–14.1)	24.2 (20.0–28.9)	39 (33.3–44.9)	33.3 (18.7–52.0)	23.6 (21.1–26.3)

**Current smokeless tobacco**	3.3 (1.9–5.8)	2.5 (1.3–4.8)	5.5 (3.3–9.0)	3.3 (0.4–20.8)	3.6 (2.6–5.0)

**Current alcohol use**	9 (6.5–12.5)	16.4 (12.9–20.7)	17.2 (13.1–22.2)	0.0 (0.0)	13.5 (11.6–15.8)

**Hypercholesterolemia**	13.9 (10.7–17.9)	19.4 (15.6–23.8)	24.3 (19.5–29.7)	16.7 (7.0–34.7)	18.7 (16.3–21.1)

**Overweight**	38.6 (33.7–43.8)	33.4 (28.7–38.5)	29.8 (24.6–35.5)	30 (16.2–48.9)	34.1 (31.3–37.2)

**Obesity**	11.1 (8.2–14.8)	12.4 (9.3–16.2)	13.2 (9.7–17.8)	13.3 (5.0–31.0)	12.2 (10.3–14.3)

**Hypertension**	29.4 (24.9–34.4)	41.6 (36.5–46.8)	44.5 (38.6–50.5)	86.7 (68.0–95.2)	39.4 (36.4–42.4)

**Diabetes mellitus**	3.9 (2.3–6.5)	8.7 (6.2–12.1)	9.2 (6.3–13.3)	20 (9.1–38.3)	7.5 (6.0–9.2)


Of all study participants, 23.6% (95% CI 21.1–26.3%) were smokers. Proportion of smoking was higher among males (42.9%) than females (8.1%) and similarly, smokeless tobacco use was higher among males (7.5%). More than one third of the total participants were overweight (34.2%, 95% CI 31.3–37.2%) and obese being 12.2% (95% CI 10.3–14.3%). Proportion of obesity among females was 14.5% while it was 9.3% among males. Prevalence of self-reported hypertension was 10.4% (95% CI 8.7–12.4%) while participants who had high blood pressure during the data collection period was 34.6% (95% CI 31.7–37.6%). Prevalence of self-reported diabetes was 2.6% (95% CI 1.7–3.7%) while participants with raised random blood glucose at the time of study was 7.1% (95% CI 5.6–8.8%). Prevalence of hypercholesterolemia was 18.7% (95% CI 16.4–21.2%) (Tables 1 and 2).

**Table 2:** Distribution of CVD risk factors of study participants by sex (N = 1018).


VARIABLES	MALE (n = 452, 44.4%) n (%)	FEMALE (n = 566, 55.6%) n (%)	TOTAL (n = 1018) n (%)	p-VALUE

**Current smoking**	194 (42.9)	46 (8.1)	240 (23.6)	<0.001

**Current smokeless tobacco use**	34 (7.5)	3 (0.5)	37 (3.6)	<0.001

**Current alcohol use (30 days)**	135 (29.9%)	1 (0.2)	136 (13.5%)	<0.001

**Overweight**	161 (35.6)	187 (33.0)	348 (34.2)	0.07

**Obesity**	42 (9.3)	82 (14.5)	124 (12.2)	0.07

**High blood pressure**	173 (38.3)	179 (31.6)	352 (34.6)	0.03

**Known case of Hypertension**	21 (4.6)	85 (15.0)	106 (10.4)	<0.001

**Raised blood glucose**	33 (7.3)	36 (6.4)	69 (6.8)	0.61

**Known case of Diabetes mellitus**	10 (2.2)	16 (2.8)	26 (2.6)	0.54

**Hypercholesterolemia**	75 (16.6)	115 (20.3)	190 (18.7)	0.13


### 3.1 Risk stratification and concordance between the two charts

Out of 1,018 participants, low CVD risk (<10%) was observed in 958 (94%) participants by laboratory-based chart and 956 (93.9%) participants by non-laboratory-based chart. Details of moderate- and high-risk assessment is described in Table 3.

**Table 3:** Agreement level between WHO Cardiovascular Disease risk prediction charts with laboratory and non-laboratory charts: (N = 1018).


	RISK LEVELS OF WHO LABORATORY-BASED CHART					

	<5% n (%)	5–9% n (%)	10–19% n (%)	20–29% n (%)	≥30% n (%)	TOTAL n

**Risk levels of WHO non-laboratory based chart**						

**<5%**	645 (63.4)	40 (3.9)	1 (0.1)	0 (0.0)	0 (0.0)	686 (67.4)

**5–9%**	68 (6.7)	175 (17.2)	27 (2.6)	0 (0.0)	0 (0.0)	270 (26.5)

**10–19%**	1 (0.1)	28 (2.8)	28 (2.8)	3 (0.3)	0 (0.0)	60 (5.9)

**20–29%**	0 (0.0)	1 (0.1)	1 (0.1)	0 (0.0)	0 (0.0)	2 (0.2)

**≥30%**	0 (0.0)	0 (0.0)	0 (0.0)	0 (0.0)	0 (0.0)	0 (0.0)

**Total**	714 (70.1)	244 (24)	57 (5.6)	3 (0.3)	0 (0.0)	1018 (100)

**CHARACTERISTICS OF CONCORDANCE**		**n (%)**				

Concordance		848 (83.3)				

Non-concordance		170 (16.7)				

Underestimate		71 (7)				

Overestimate		99 (9.7)				

K value-Cohen’s Kappa (95% CI)		Agreement = 83.3%Expected agreement = 53.9%kappa – value = 0.64 (0.02)				


The concordance between the laboratory-based and non-laboratory-based charts in CVD risk classification of the participants into different risk categories was 848 (83.3%). Among the 170 participants with non-concordant risk estimates, 71 (58.2%) were overestimates and 99 (41.8%) were underestimates. Majority (98.8%) of the non-concordant belonged to risk categories <20%. The Cohen’s kappa between the two charts was 0.64 (SE 0.02), reflecting good agreement. Considering lab-based charts considered as the gold standard, the sensitivity and specificity of non-lab-based charts was 86.5% and 90.3% respectively (Table 3).

### 3.2 Sensitivity analysis

As the risk charts are meant to be used for people in the age group of 40–74 years, we did a sensitivity analysis by removing participants in the age group of 30–39 years. The concordance observed was 78.1% (514/658) between laboratory and non-laboratory-based risk charts. Under-estimation by non-laboratory-based charts was 8.7% (57/658) while over-estimation was 13.2% (87/658). The kappa statistic between the two also slightly decreased to 0.61 from previous value of 0.64, but it still indicates good agreement between the two charts.

## 4 Discussion

Drug therapy and counselling for individuals with high risk of developing fatal and non-fatal cardiovascular disease (CVD) is a World Health Organization (WHO) best-buy intervention. Risk prediction charts help identify high risk individuals and many tools have been developed over the years. Although many studies have been published on CVD risk estimate using various risk prediction charts including WHO/ISH risk charts, the present study is one of the first to estimate CVD risk using the 2019 updated WHO CVD risk charts since its development.

Prevalence of current tobacco use in the present study was 25.7% as compared to 32.8% in National Non-communicable disease survey (NNMS) 2017–18 and 28.6% in Global Adults Tobacco Survey-2(GATS-2) but tobacco use (smoked) is higher in the present study due to the widespread use of hookah in the study area [12,16]. The prevalence of alcohol consumption (13.6%) is comparable to that found in a study of substance use in India survey (14.6%) and NNMS (15.9%) [12,17]. Smoking and alcohol consumption was higher in men. The prevalence of overweight and obesity found was 34.2% and 12.2% respectively which was much higher than that found in NNMS 2017–18, 26.1% being overweight and 6.2% being obese. Obesity was higher among women. The prevalence of raised blood pressure was 34.6% while the same was 28.5% in NNMS 2017–18 and raised blood glucose was 6.8% as compared to 9.3% in NNMS 17–18.

In the present study, 94% had CVD risk <10% estimated by both the charts. It was observed that there was a high concordance of 83.3% between the laboratory-based and non-laboratory-based CVD risk charts. The 2019 WHO CVD risk charts have been recently developed and until now, there are no published studies assessing the concordance between the laboratory based and non-laboratory based WHO CVD risk charts. There are previous studies assessing the concordance of WHO/ISH risk prediction tool between the charts with cholesterol and without cholesterol levels, which reported concordance in the range of 86.3% to 89.5%. A study by Deori et al. (2020) observed a concordance of 86.3% while concordance of 89.5% and 88.1% was observed by Fatema et al. (2015) and Nordet et al. (2013) respectively [13,14,15]. In our study, the kappa statistic between the two WHO CVD risk charts was 0.64. The kappa statistic shows a good agreement between the two charts in 30–69 years age group and a concordance of 83.3% suggests that non-laboratory-based risk charts can be a useful tool in resource limited settings. Also, as non-laboratory-based risk charts do not require laboratory investigations for diagnosis of diabetes mellitus and total cholesterol measurement, it can be used by the community health workers to predict the CVD risk estimate of individuals at community level. According to the guidelines, individuals with a total CVD risk level of 10% and above should receive an assessment using laboratory-based charts after measurement for diabetes and cholesterol.

This was one of the first studies to assess the CVD risk using the updated 2019 WHO CVD risk charts to determine the concordance between the lab-based and non-lab based WHO CVD risk charts at community level setting. There were some limitations of the study. Firstly, we included participants aged below 40 years, although the charts are applicable to 40 years and above. Since the younger population is expected to have lower level of CVD risk, and exclusion of persons above 69 years, the study may have underestimated the level of CVD risk in this population. The reason for including people aged 30 and above is because the guidelines under National Health Mission in India recommends screening from the age of 30 years. However, a sensitivity analysis excluding the participants in the age group of 30–39 years showed that the concordance was comparable to the concordance found in the overall study population. Secondly, use of random blood glucose measurement by point of care device to diagnose diabetes mellitus may have led to a misclassification of people with diabetes.

## 5 Conclusion

The present study highlighted that there is low CVD risk using WHO CVD risk chart among adults in rural community in north India. Good agreement between the WHO CVD laboratory-based and non-laboratory-based risk charts was observed in the study. Thus, non-laboratory-based charts could be considered for identifying the individuals who might benefit from laboratory-based risk assessment. A risk stratification approach is particularly suitable to settings with limited resources, where saving the greatest number of lives at lowest cost becomes imperative. A stepwise approach can be considered starting with non-lab risk assessment and followed by laboratory-based risk; this will help in optimizing resources. Additionally, the non-laboratory risk charts can be used for education and advocacy regarding total CVD risk in areas where lab testing remains currently unavailable.

## Data Accessibility Statement

Dataset associated with the current paper is available from the corresponding author upon reasonable request.
